# Confessions of a Neurasthenic

**Published:** 1910-07

**Authors:** 


**Confessions of a Neurasthenic.** By **William Taylor Marrs,** M.D. 12 mo, pp. 120. Philadelphia: F. A. Davis Co. (Cloth, $1.00.)

If you have not yet read "Confessions of a Neurasthenic" get the manual and read every word. The book is written in a half humorous vein, but makes points all along the line. As a "chaser of the blues" it is well worth reading. Between the lines and only partially hidden is a vast amount of good sense.

